# SCORE2 Screening Tool for Cardiovascular Risk Assessment in Psoriasis—A Case–Control Study

**DOI:** 10.3390/jcm13113237

**Published:** 2024-05-30

**Authors:** Tomáš Kampe, Janette Baloghová, Peter Kolarčik, Kvetoslava Rimárová, Erik Dorko

**Affiliations:** 1 Department of Dermatovenerology, Faculty of Medicine, University Hospital, P. J. Safarik University, 040 01 Kosice, Slovakia; tomas.kampe@upjs.sk (T.K.); janette.baloghova@upjs.sk (J.B.); 2 Department of Health Psychology and Research Methodology, Faculty of Medicine, P. J. Safarik University, 040 01 Kosice, Slovakia; peter.kolarcik@upjs.sk; 3 Olomouc University Social Health Institute, Palacky University Olomouc, 779 00 Olomouc, Czech Republic; 4 Department of Public Health and Hygiene, Faculty of Medicine, P. J. Safarik University, 040 01 Kosice, Slovakia; kvetoslava.rimarova@upjs.sk

**Keywords:** atherosclerosis, cardiovascular disease, cardiovascular risk, Framingham risk score, psoriasis, SCORE2 risk score

## Abstract

**Background**: Psoriasis is a common, T-cell-mediated inflammatory and immune-mediated skin disease. Numerous studies confirmed that patients with psoriasis have a significant frequency of cardiovascular (CV) risk factors and CV diseases (CVDs). Risk stratification is helpful in light of the elevated risk of CVD in psoriasis patients. SCORE2 and SCORE2-OP, a new algorithm derived, calibrated and validated to predict the 10-year risk of first-onset CVD in European populations, enhances the identification of individuals at higher risk of developing CVD across Europe. **Objective**: Using the SCORE2 and SCORE2-OP scoring systems, the current study objective was to evaluate CV risk in Slovak psoriasis patients and the relationship between CV risk and psoriasis features in a real-world setting. **Results**: A case–control study was conducted involving 115 outpatients with plaque psoriasis and 66 age- and gender-matched controls with skin conditions other than psoriasis. Patients with psoriasis had significantly higher mean SCORE2 values. In the age group up to 50 years, more psoriasis patients were classified as moderate risk than controls (33.8% vs. 13.6%, *p* = 0.010); the high-risk category was dominated by psoriasis patients. Analysing the relationship between CV risk and selected variables, we determined, using linear regression, the dependence of the SCORE2 risk score on gender in the age group up to 50 years, on age in both age groups, on waist circumference (WC) in the category up to 50 years and on the duration and severity of psoriasis in both age groups using linear regression. For individuals older than 70, we estimated the SCORE2-OP risk score, with the average risk score being 19.5 ± 4.95. We did not observe controls with a high risk score. Psoriasis patients were more likely to be smokers and had significantly higher mean values for body mass index (BMI), WC, total cholesterol (TC), low-density lipoprotein (LDL) and systolic blood pressure (BP). **Conclusions**: Because CV risk factors and psoriasis are strongly related, the importance of CV risk stratification is growing, and initiating preventive lifestyle changes or therapeutic interventions in patients with psoriasis is warranted.

## 1. Introduction

Psoriasis is a systemic inflammatory disorder that involves complex pathogenic interactions between the innate and adaptive immune systems; it affects approximately 2% of the population [1]. Psoriasis increases the relative risk of atherosclerosis (AS) independent of traditional risk factors such as smoking, obesity and dyslipidaemia, according to accumulating evidence [2]. Of developing concern is the connection to cardiovascular diseases (CVDs) deriving from AS, as this adds greatly to higher patient mortality [3]. Risk stratification for CVD is used for both the primary and secondary prevention of fatal and non-fatal cardiovascular (CV) events. Predicting a patient’s CV risk is essential for improving CV prevention efforts, even in those without overt CVD or diabetes. Psoriasis patients are at a higher risk of CVD; therefore, identifying and stratifying patients according to their risk for CVD should be an intrinsic aspect of patient management [4].

Unlike the general population and individuals with diabetes, rheumatoid arthritis and systemic lupus, there are very few data on CV risk estimation using predictive calculators in psoriasis patients [5]. Recent investigations have assessed the risk of CV events in psoriasis patients using the Framingham Risk Score (FRS) [6], although it is recognised that the FRS tends to overestimate absolute risk in populations with a low incidence of ischemic heart disease [7]. European professional societies developed the SCORE system (Systematic Coronary Risk Evaluation system) to correct and estimate the level of risk for the European population. This system is based on knowledge of blood pressure values, total cholesterol (TC) levels and smoking status, as well as gender, patient age and belonging to a country with a different level of CV risk within Europe [8]. However, SCORE only covers fatal CVD events; thus, it underestimates the total CVD burden, which has changed in recent decades towards non-fatal outcomes, particularly among younger individuals. SCORE does not account for considerable differences in risk between countries within the same risk region, so it may overestimate risk in these circumstances [9]. Consequently, SCORE2, an updated algorithm tailored to European populations to predict the 10-year risk of first-onset CVD, was developed in individuals without previous CVD or diabetes aged 40–69 years (age subcategories under 50 years and 50 to 69 years) [4] and SCORE2-OP in individuals over 70 years of age in four geographical risk regions of Europe [10]. Individuals with diabetes are typically thought to be at a high risk for CVD, and risk ratings for this population already exist [11]. SCORE2 outperformed SCORE by avoiding an overestimation of risk and correctly categorising persons with a greater observed lifetime CVD risk as high risk. Each of the groupings has a distinct SCORE2 and SCORE2-OP nomogram. For Slovakia, a high-risk model was established [8].

**Figure 1:**
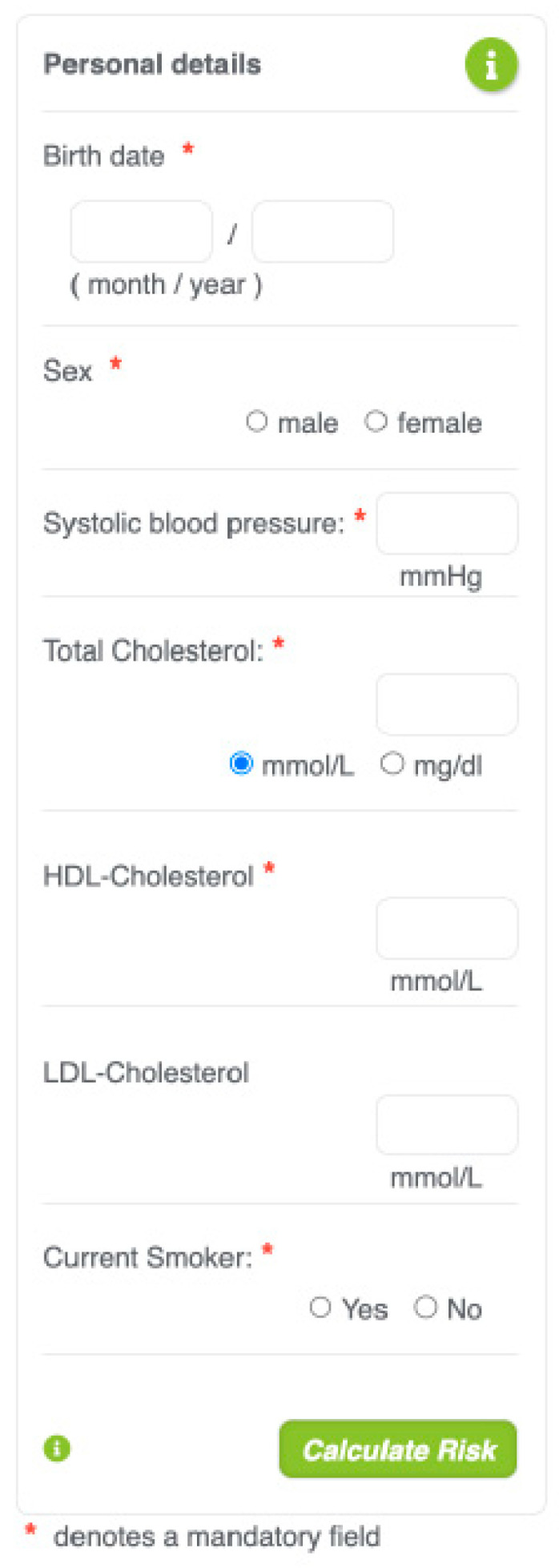
Online SCORE2 calculator.

In order to compute CV risk utilising SCORE2 or SCORE2-OP through the online calculator, plasma concentrations of total cholesterol and HDL-cholesterol are required. Although LDL-C measurement is mandatory, it is advised by guidelines. Additional required data consist of age, gender, systolic blood pressure, and smoking habit (Figure 1).

The purpose of this study was to evaluate CV risk in Slovak psoriasis patients and the relationship between CV risk and psoriasis features in a real-world setting using the SCORE2 and SCORE2-OP scoring systems.

## 2. Materials and Methods

In a monocentric case–control study conducted in the dermatology outpatient department (University Hospital, Kosice, Slovakia) between November 2021 and May 2022, consecutive patients older than 18 years with a clinically confirmed diagnosis of plaque psoriasis were included. The control group consisted of non-psoriatic patients (patients with melanocytic nevi, non-serious skin infections and other benign disorders) from the same department. Each participant supplied written informed consent, and the regional ethics council for clinical research authorised the study. Patients and controls with any of the following were excluded: (a) history of CVD, (b) history of stroke, (c) history of diabetes, (d), age under 18 years, (e) pregnant or lactating women. In particular, parameters collected for each subject were age, gender, TC, high-density lipoprotein (HDL), LDL, systolic BP and smoking status. Other data collected consisted of BMI and WC. Arterial hypertension, CVD and diabetes were identified in the personal history. The kind of therapy, age of psoriasis onset and psoriasis severity were evaluated in individuals with psoriasis using the Psoriasis Area and Severity Index (PASI) score. Psoriasis with a PASI score < 10 was regarded as mild, whereas psoriasis with a PASI > 10 was deemed moderate to severe. Patients were categorised as having early-onset psoriasis if the age of onset was before 40 years old (type I psoriasis).

Each subject’s 10-year CV risk was estimated using the SCORE2 scoring system (for individuals aged 40 to 69 years) and the SCORE2-OP scoring method (for individuals 70 years or older) in individuals without a history of CVD and diabetes. For computing the risk score, a logistic function was employed, which included the individual’s age, gender, current systolic blood pressure, smoking status and levels of TC, LDL and HDL. A free SCORE2 and SCORE2-OP calculator is accessible at http://www.escardio.org/ (accessed on 8 February 2022).

Initially, a descriptive analysis of the data was performed using frequency tables. We determined the absolute and relative prevalence of important categorical variables, as well as the means and standard deviations (SD) of relevant continuous variables. We also evaluated potential subgroup differences using Pearson’s chi-squared test for categorical variables and the Mann–Whitney U-test for continuous variables. After describing the sample, we examined the relationship between independent variables and the outcome variable, SCORE2/SCORE2-OP. We employed a linear regression analysis to identify pertinent predictors. All statistical analyses were conducted with the aid of the statistical program IBM SPSS 23.0.

## 3. Results

The study included 115 patients and 66 controls. Of the psoriasis patients, 29 (25.2%) had mild psoriasis and 86 (78.9%) had moderate to severe psoriasis; the mean PASI was 14.21 ± 6.42. Psoriasis duration was 1 to 57 years (mean 15.90 ± 11.34), and 58.3% of patients had type I psoriasis. Psoriasis patients were more likely to be smokers and had significantly higher mean values of BMI, WC, TC levels, LDL values and systolic blood pressure than the control group, and 73.7% of patients had received biological treatment. Table 1 contains descriptive assessments of the demographic, psoriasis and CVD risk factor variables.

In both age groups, patients with psoriasis had significantly higher mean SCORE2 values (mean ± SD, 2.55 *±* 2.38 versus 1.66 *±* 1.00, *p* = 0.097 and 8.25 *±* 4.48 versus 6.75 *±* 5.31, *p* = 0.063, respectively) (Table 2). In the age group up to 50 years, more psoriasis patients were classified as moderate risk than controls (33.8% vs. 13.6%, *p* = 0.010), with 5.4% of psoriasis patients falling into the high-risk category. We discovered moderate risk in 65.8% of patients and 90.9% of controls between the ages of 50 and 69. The high-risk category was dominated by psoriasis patients (31.6%). For individuals older than 70, we estimated the SCORE2-OP risk score. The average risk score was 19.5 ± 4.95. We did not observe any controls with a high risk score (Table 3). In the age category of up to 50 years, we detected a difference between the genders in the average SCORE2 risk score of patients with psoriasis and controls, but the difference was greater in the psoriasis group. Men between the ages of 50 and 69 had a higher risk score, but the difference was not statistically significant. We demonstrated the influence of the severity and duration of psoriasis on the CV score for both age groups (Table 4). Using linear regression, we determined the dependence of the risk score on gender in the age category up to 50 years, on age in both categories, on WC in the category up to 50 years and on the duration and severity of psoriasis in both categories when analysing the connection between CV risk and the chosen variables. (Table 5).

## 4. Discussion

According to our knowledge, this is the first study undertaken in Slovakia to analyse the CV risk profile of psoriasis patients. CVDs are the most common fatal non-communicable diseases globally, remaining a major cause of morbidity and mortality in Europe. Heart and blood vessel diseases continue to be the leading cause of death in the Slovak Republic; we rank last among EU member states in this regard. The European Society of Cardiology (ESC) provides guidelines and advocates the use of risk prediction models to enhance healthcare and population-wide prevention [12]. Typically, risk models that combine information on numerous conventional CVD risk variables predict an individual’s 10-year risk. The objective is to identify those at increased risk for CVD who would benefit most from preventative measures. However, the estimate of CV risk in psoriasis patients using conventional ratings has significant drawbacks. These predictive techniques were not designed specifically for patients with inflammatory disorders such as psoriasis, and their performance is suboptimal, because traditional CV risk variables do not adequately explain the increased CV risk in individuals with psoriasis. Current risk algorithms do not account for systemic inflammation, a critical factor in AS. Thus, the CV risk in psoriasis is frequently underestimated [5,13,14]. Some proposals for CV risk management suggested applying a 1.5-times multiplier to any derived CV risk score to account for these restrictions. Adding an additional predictive feature, such as the diagnosis of subclinical AS, is another option to maximise risk stratification [15].

Most epidemiological studies of CV risk have relied on the FRS, which is regarded to be overestimated in countries with a lower frequency of CV events [7,16,17,18]. In addition to the FRS, one study estimated CV risk using SCORE, DORICA and REGICOR, which are better suitable for a Mediterranean population [5]. Several studies have examined the relationships between FRS, age and the severity of psoriasis. At 5 years (mean ± SD: 5.3 ± 4.4 vs. 3.4 ± 3.3, *p* < 0.001) and 10 years (11.2 ± 8.1 vs. 7.3 ± 6.3, *p* < 0.001), Gisondi et al. [16] observed that the FRS in psoriasis patients was considerably greater than in controls. At 5 and 10 years, patients aged 50 to 60 years had an absolute risk of experiencing a major CV event of 5.3% and 11.2%, respectively, which was deemed an intermediate risk. According to the FRS, Rosa et al. [17] identified low risk, intermediate risk and high risk in 68.4%, 18.4% and 13.3% of patients, respectively. Kimball et al. [18] computed risk ratings for 2.899 individuals participating in three phase III ustekinumab clinical studies. More than half of patients with moderate to severe psoriasis had at least two CV risk factors, and according to the FRS, 12.3% of patients were at moderate risk and 18.6% were at high risk. According to research by Eder et al. [14], the FRS has a limited ability to appropriately stratify psoriasis patients. After ultrasound evaluation of the existence of subclinical AS, the majority of patients in the intermediate-risk group and over half of patients in the low-risk group based on this score were reclassified into the higher-risk group. In individuals with psoriatic arthritis, risk underestimation was even greater. Another study that analysed many risk levels categorised the majority of psoriasis patients as low risk [5]. Patients with psoriasis did not have an elevated risk of coronary heart diseases, according to research by Gyldenlve et al. [19]. In our study, we calculated the SCORE2 and SCORE2-OP scores, which had not been previously used for psoriasis patients and are more suited to the demographics and high-risk area of Europe. Consistent with previous research, we found that psoriasis patients had a higher CV risk score than controls. In the age group up to 50 years, more psoriasis patients were classified as moderate risk than controls (33.8% vs. 13.6%, *p* = 0.010); however, in the age group 50 to 69 years, the moderate risk was higher in the control group. Psoriasis patients dominated the high-risk category.

Psoriasis was independently related to a higher FRS in an Italian study; however, there was no association between psoriasis severity or duration and FRS [16]. Nearly three-quarters of intermediate- and high-risk patients had a PASI ≤ 10, according to Rosa et al. [17]. This association was not investigated in the remaining papers provided, though Doukaki et al. made comparable observations [20]. Previous research has revealed that psoriasis and CVD interact with age, with younger individuals having a much higher relative risk of CVD than older patients [21]. Analysing the relationship between CV risk and selected variables, we determined the dependence of the risk score on gender in the age category up to 50 years, on age in both age categories, on WC in the category up to 50 years and on the duration and severity of psoriasis in both categories using linear regression. These risk estimates may reflect the bimodal incidence of psoriasis as well as the differential effect of early-onset psoriasis on the course of AS. Alternately, the development of additional CVD risk-factors that correspond with age may eventually exceed the added CVD risk posed by psoriasis. Recent studies have indicated, however, that patients with psoriasis who undergo coronary angiography are more likely to have coronary artery disease among senior cohorts [22]. In our study, a greater proportion of patients under 50 years of age with psoriasis were classified as moderate risk than controls. This age range represents a common age at which CV risk factor interventions can substantially modify future CV risk.

As CVD and psoriasis share comparable risk factors, it is critical that physicians conduct concurrent screenings for both conditions in their patients. It is imperative that individuals diagnosed with psoriasis recognise that their risk factor profile confers an elevated susceptibility to cardiovascular disease. To mitigate the likelihood of developing heart disease, they ought to make lifestyle modifications. In addition to the treatment of skin manifestations, the practice of dermatology incorporates comprehensive patient care. As a result of infrequent access to primary care physicians, psoriasis patients frequently consult dermatologists as their primary healthcare provider. Nevertheless, awareness programs and routine preventive assessments are insufficient to achieve this objective. Subsequently, it is essential to obtain risk factor data from every preventive visit conducted at a general practitioner. This information will be utilised to calculate the CV risk of individual patients over the subsequent decade. More precisely, it will be used to communicate to the insured the manner in which alterations in individual risk factors will impact their overall risk. It is strongly recommended that dermatologists conduct annual examinations for cardiovascular risk factors, including assessments of hypertension, diabetes, and dyslipidaemia. For cases of moderate to severe psoriasis, screening should occur earlier and more frequently. A multidisciplinary approach involving primary care providers and preventive cardiologists is emphasised. Cessation of smoking, the effective management of blood pressure, and lipid-lowering treatments, particularly statins, reduce CV morbidity and mortality by a substantial margin. As a result of their pleiotropic anti-inflammatory properties, statins have emerged as potential treatments for psoriasis symptoms as well as cardiovascular disease risk reduction [23]. An estimate of total CV risk is a component of a continuum. The criteria utilised to establish high-risk status are, to some extent, subjective and predicated on the risk thresholds beyond which clinical trials demonstrate benefit. Consideration should be given to practical issues pertaining to the local healthcare systems in clinical practice. Aside from identifying and managing those at high risk, those at moderate risk should also be counselled by experts on how to modify their lifestyles; in certain instances, pharmacotherapy may be required to reduce the risk of atherosclerosis.

People at low risk should be provided with guidance to help them maintain their status. Therefore, preventive measures should be tailored to the patient’s total CV risk in terms of intensity. SCORE calculators could help us reduce morbidity and mortality in the overall population, but especially in a very important group of patients—asymptomatic high-risk individuals with psoriasis.

## 5. Conclusions

The majority of epidemiological and clinical-translational data indicate a substantial contribution of psoriasis to CVD when psoriasis is viewed as a risk factor for CVD, regardless of its severity. Consideration should also be given to patients with a significant burden of psoriatic disease (long duration or long-term untreated) and an elevated CV risk. The identification of CV risk using easy and inexpensive techniques is extremely crucial. SCORE2 and SCORE2-OP, new algorithms derived, calibrated and validated to predict the 10-year risk of first-onset CVD in European populations, improve the identification of persons at increased risk of developing CVD in Europe. The SCORE2 assessment is also a component of the Ministry of Health of the Slovak Republics initiative to reduce the occurrence of avoidable CVD through the systematic monitoring of CV risk in a primary care setting. Persons with chronic inflammatory illnesses, such as psoriasis, are also candidates for the systematic CV risk monitoring programme.

## Figures and Tables

**Table 1:** Characteristics of the study population.

Characteristics	Psoriasis Patients	Controls	Difference Testing	
	(n = 115)	(n = 66)	U/chi2 Value	*p* Value
Gender				
Male, %, n	51.3% (59)	39.4% (26)	2.388	0.122
Age (years), mean ± SD	43.69 ± 13.31	45.51 ± 11.73	3434.000	0.287
Current smoker, %, n	40.0 (46)	30.3 (20)	8.445	0.015
TC (mmol/L), mean ± SD	5.60 ± 1.14	4.72 ± 1.00	2127.500	<0.001
LDL (mmol/L), mean ± SD	2.72 ± 1.07	2.48 ± 0.81	3289.500	0.136
HDL (mmol/L), mean ± SD	1.31 ± 0.34	1.35 ± 0.30	3498.000	0.381
Systolic blood pressure (mmHg), mean ± SD	127.15 ± 10.72	121.42 ± 10.11	2537.500	<0.001
BMI (kg/m^2^)	25.48 ± 4.59	24.84 ± 4.70	3432.000	0.285
WC (cm)	87.09 ± 12.29	82.50 ± 10.52	2959.00	0.014
Psoriasis duration (years), mean ± SD	15.90 ± 11.34			
Range	1–57			
PASI, mean	14.21 ± 6.42			
Range	0–22			
PASI < 10, %, n	25.2 (29)			
PASI > 10, %, n	78.9 (86)			
Psoriasis type I, %, n	58.3 (67)			
Psoriasis type II, %, n	19.1 (22)			
Systemic non-biological therapy, %, n	23.68 (27)			
Systemic biological therapy, %, n	73.68 (84)			
Other therapy, %, n	3.48 (4)			

**Table 2:** Comparison of mean SCORE2 and SCORE2-OP in patients with psoriasis and controls.

	Psoriasis Patients		Controls		Difference Test	
Age Group	n	Mean ± SD	n	Mean ± SD	U Value	*p* Value
<50 years	75	2.55 ± 2.38	44	1.66 ± 1.00	1347.000	0.097
50–69 years	38	8.25 ± 4.38	22	6.75 ± 5.31	297.000	0.063
>70 years	2	19.5 ± 4.95	0			

**Table 3:** Comparison of SCORE2 risk categories in patients and controls.

Risk Category	Psoriasis Patients	Controls	Difference Testing	
	%, n	%, n	Chi2 Value	*p* Value
Age < 50 years				
Low risk <2.5%	60.8 (45)	86.4 (38)		0.010
Moderate risk 2.5 to <7.5%	33.8 (28)	13.6 (6)	9.203	
High risk ≥7.5%	5.4 (4)	0.0 (0)		
Age 50–69 years				
Low risk <2.5%	2.6 (1)	0.0 (0)		0.092
Moderate risk 5–10%	65.8 (25)	90.9 (20)	4.771	
High risk ≥10%	31.6 (12)	9.1 (2)		

**Table 4:** Comparison of SCORE2 and SCORE2-OP values between patients and controls in age categories and between groups according to sex, PASI and type of psoriasis.

		Psoriasis Patients		Difference Test		Controls		Difference Test	
		n	Mean *±* SD	U Value	*p* Value	n	Mean *±* SD	U Value	*p* Value
Gender									
<50 years	male	40	3.11 ± 2.29	369.500	<0.001	17	1.85 ± 0.95	153.500	0.046
	female	35	1.90 ± 2.34			27	1.54 ± 1.04		
50–69 years	male	18	9.22 ± 3.68	121.500	0.087	9	5.61 ± 2.80	52.500	0.688
	female	20	7.38 ± 4.86			13	7.54 ± 6.52		
>70 years	male	1	16.00	0.000	0.317	0			
	female	1	23.00			0			
PASI									
<50 years	PASI < 10	17	1.464 ± 0.8652	242.500	0.21				
	PASI > 10	57	2.821 ± 2.5997						
50–69 years	PASI < 10	11	5.875 ± 4.1469	67.500	0.73				
	PASI > 10	29	8.897 ± 4.3597						
>70 years	PASI < 10	2	23.000						
Psoriasis type									
<50 years	type I	52	3.24 ± 2.68	27.000	0.343				
	type II	23	1.50 ± 0.71						
50–69 years	type I	20	8.37 ± 5.12	169.500	0.964				
	type II	18	7.86 ± 3.53						
>70 years	type I	0							
	type II	2	19.50 ± 4.95						

**Table 5:** Associations of selected predictors with SCORE2 levels, analysed using linear regression and stratified by patient and control subgroup.

		Psoriasis Patients		Control	
	Age Category	B (95% CI)	*p* Value	B (95% CI)	*p* Value
Gender	<50 years	−1.22 (−2.29|0.14)	0.27	−0.32 (−0.94|0.31)	0.315
	50–69 years	1.85 (−4.71|0.01)	0.198	1.93 (−2.91|6.77)	0.416
Age	<50 years	0.13 (0.06|0.19)	<0.001	0.02 (−0.03|0.06)	0.448
	50–69 years	0.56 (0.37|0.74)	<0.001	0.29 (−0.12|0.71)	0.157
BMI	<50 years	0.09 (−0.03|0.21)	0.142	0.04 (−0.04|0.12)	0.295
	50–69 years	0.06 (−0.27|0.39)	0.708	0.11 (−0.32|0.55)	0.596
WC	<50 years	0.05 (0.00|0.09)	0.036	0.01 (−0.03|0.04)	0.698
	50–69 years	0.06 (−0.07|0.19)	0.376	−0.10 (−0.32|0.13)	0.372
Psoriasis	<50 years	0.05 (−0.02|0.12)	0.164		
duration	50–69 years	0.12 (0.03|0.21)	0.012		
PASI	<50 years	0.10 (0.01|0.19)	0.037		
	50–69 years	0.23 (0.01|0.45)	0.044		

## Data Availability

Data are contained within the article.
